# The Complex Interplay between Arbuscular Mycorrhizal Fungi and Strigolactone: Mechanisms, Sinergies, Applications and Future Directions

**DOI:** 10.3390/ijms242316774

**Published:** 2023-11-26

**Authors:** Gökhan Boyno, Younes Rezaee Danesh, Semra Demir, Necmettin Teniz, José M. Mulet, Rosa Porcel

**Affiliations:** 1 Department of Plant Protection, Faculty of Agriculture, Van Yuzuncu Yil University, Van 65090, Türkiye; 2 Department of Plant Protection, Faculty of Agriculture, Urmia University, Urmia 5756151818, Iran; 3 Department of Agricultural Biotechnology, Faculty of Agriculture, Van Yuzuncu Yil University, Van 65090, Türkiye; 4 Instituto de Biología Molecular y Celular de Plantas, Universitat Politècnica de València-Consejo Superior de Investigaciones Científicas, 46022 Valencia, Spain

**Keywords:** arbuscular mycorrhizal fungi, strigolactone, synergistic interaction, AM symbiosis, sustainable agriculture

## Abstract

Plants, the cornerstone of life on Earth, are constantly struggling with a number of challenges arising from both biotic and abiotic stressors. To overcome these adverse factors, plants have evolved complex defense mechanisms involving both a number of cell signaling pathways and a complex network of interactions with microorganisms. Among these interactions, the relationship between symbiotic arbuscular mycorrhizal fungi (AMF) and strigolactones (SLs) stands as an important interplay that has a significant impact on increased resistance to environmental stresses and improved nutrient uptake and the subsequent enhanced plant growth. AMF establishes mutualistic partnerships with plants by colonizing root systems, and offers a range of benefits, such as increased nutrient absorption, improved water uptake and increased resistance to both biotic and abiotic stresses. SLs play a fundamental role in shaping root architecture, promoting the growth of lateral roots and regulating plant defense responses. AMF can promote the production and release of SLs by plants, which in turn promote symbiotic interactions due to their role as signaling molecules with the ability to attract beneficial microbes. The complete knowledge of this synergy has the potential to develop applications to optimize agricultural practices, improve nutrient use efficiency and ultimately increase crop yields. This review explores the roles played by AMF and SLs in plant development and stress tolerance, highlighting their individual contributions and the synergistic nature of their interaction.

## 1. Introduction

Plants are pivotal for many ecosystems and thus essential for the survival of virtually all living organisms. They are not only a source of food for humans and animals and the main point of entrance of solar energy and organic carbon in ecosystems, but they also play a critical role in regulating the Earth’s climate and sustaining the planet’s biodiversity. However, plants are constantly under threat from various biotic and abiotic stresses, such as pests, diseases and environmental factors, like drought and salinity [1,2,3]. Under the current context of anthropogenic global warming, forest and cultivated plants must adapt to the novel conditions or become extinct [4,5]. To overcome these challenges, plants have evolved complex mechanisms that involve a wide range of signaling pathways and interactions with other organisms, including microbes.

The significance of arbuscular mycorrhizal fungi (AMF) and strigolactones (SLs) in plant–microbe interactions lies in their ability to positively influence plant growth, development and overall health [6,7]. AMF establish mutualistic associations with plants, colonizing their root systems and providing various benefits [8]. These benefits include enhanced nutrient acquisition, such as an increased availability of phosphorus and micronutrients, improved water uptake and protection against biotic and abiotic stresses [2]. AMF can also induce systemic resistance in plants, making them more resistant to pathogens and pests and abiotic stresses [9,10]. On the other hand, SLs, a class of plant hormones, regulate several critical processes in plants [11]. They are involved in shaping root architecture, promoting the development of lateral roots and stimulating the establishment of beneficial associations with symbiotic microbes, such as AMF [7,11,12]. SLs also play a role in plant defense responses, including the activation of systemic defense mechanisms against pathogens and the induction of plant secondary metabolites [13].

Recent studies have shown that the synergistic interaction between AMF and SLs can have a significant impact on plant–microbe interactions and plant development. The combined effects of these two components result in improved plant growth, an increased resistance to biotic and abiotic stresses and enhanced nutrient uptake [6,7,14]. Therefore, understanding the interplay between these two components is crucial for developing sustainable agricultural practices and improving crop yields.

Mechanistically, AMF have been found to influence the production and release of SLs by plants [15]. They can stimulate the synthesis and secretion of SLs, which act as signaling molecules to attract beneficial microbes and promote symbiotic interactions [16]. In turn, SLs can modulate the colonization and establishment of AMF within the plant root system, facilitating their beneficial effects [6,7,14]. The significance of this synergistic interaction becomes evident in its potential to improve crop productivity, nutrient utilization and plant resistance in the face of environmental challenges [6,14]. By harnessing the combined effects of AMF and SLs, agricultural practices can be optimized to enhance nutrient acquisition efficiency, leading to increased crop yields.

In this review article, we aim to explore the importance of AMF and SLs in plant–microbe interactions and plant development. We discuss the individual roles of these components and their synergistic interaction, highlighting the benefits that can be gained by harnessing their combined effects. Our objectives are to provide a comprehensive overview of the current research in this field, identify gaps in the knowledge and suggest future directions for the research. By doing so, we hope to contribute to the development of new strategies for sustainable agriculture and plant growth promotion, a major objective in the current contexts of climate change and increasing world population.

## 2. Overview of Arbuscular Mycorrhizal Fungi

Arbuscular mycorrhizal fungi extend their hyphae into the soil, exploring a larger volume and accessing nutrients inaccessible to the root [17,18]. In addition, AMF spore dynamics are found at higher densities in rhizosphere soil [19]. They can solubilize nutrients from solid soil particles and organic matter, making them available for plant uptake [20]. Furthermore, AMF release enzymes that break down complex organic compounds, releasing nutrients for plant uptake [21]. They can convert inorganic forms of macronutrients, such as phosphorus, into molecules that plants can assimilate [22]. This promotes efficient nutrient utilization by plants. Furthermore, AMF can affect the synthesis, release and signaling pathways of growth-promoting phytohormones, such as SLs, auxins and cytokinins in plants, leading to enhanced plant growth and development [15,23].

AMF can induce systemic resistance in plants, preparing them for pathogen and pest attacks. For example, AMF activate plant defense mechanisms by triggering the expression of defense-related genes [24]. This leads to the production of defense compounds, such as pathogenesis-related proteins and antimicrobial peptides that protect plants against pathogens and pests [25]. Furthermore, AMF prime the plant’s immune system, enabling a more rapid and effective defense response upon pathogen or pest attacks [25]. This priming improves the plant’s ability to recognize and respond to subsequent challenges, increasing its overall disease resistance.

### 2.1. Molecular Signaling

AMF are beneficial soil microorganisms that form symbiotic relationships with plants, enhancing nutrient uptake and contributing to the health and sustainability of terrestrial ecosystems [2]. In the early stages of AMF symbiosis, molecular signals are exchanged between the plant and the fungus [26,27]. The plant releases signaling molecules, such as SLs, into the soil in response to nutrient stress [28]. AMF hyphae in the soil sense these signals and initiate a molecular response [7,29]. This includes the expression of genes related to hyphal growth and colonization.

The growth and branching of AMF hyphae towards plant roots are regulated by various signaling pathways [17,18]. These pathways involve receptor proteins on the fungal hyphae that recognize specific plant signals. The interaction between plant root cells and AMF hyphae involves molecular cross-talk, allowing the hyphae to penetrate root cells [30]. This process involves the exchange of signaling molecules and the activation of genes that facilitate the establishment of arbuscules, specialized structures within root cells where nutrient exchange occurs [31].

Nutrient exchange in AMF symbiosis is highly dependent on molecular processes. Within arbuscules, specific transporters and channels facilitate the movement of nutrients between the plant and the fungus [7,28]. The plant provides sugars obtained by photosynthesis and organic compounds from the plant’s own metabolic pathways through molecular transporters, while the fungus supplies the plant with essential nutrients, like phosphorus and nitrogen, which the fungi have solubilized from the soil [32,33]. AMF also influence plant defense and stress responses at the molecular level [9]. They can enhance the plant’s ability to withstand various environmental stresses, such as biotic and abiotic [2,10]. The molecular mechanisms involved in these responses include the activation of defense-related genes and the modulation of plant hormone signaling pathways [9,34]. The molecular interactions between plants and AMF are highly intricate and involve the exchange of signaling molecules, gene regulation and the coordination of various molecular processes to establish and maintain this symbiotic relationship [34,35]. The synergy between the two organisms at the molecular level results in improved nutrient uptake and enhanced plant resilience.

### 2.2. Mycorrhizal Symbiosis Genes

**Figure 1:**
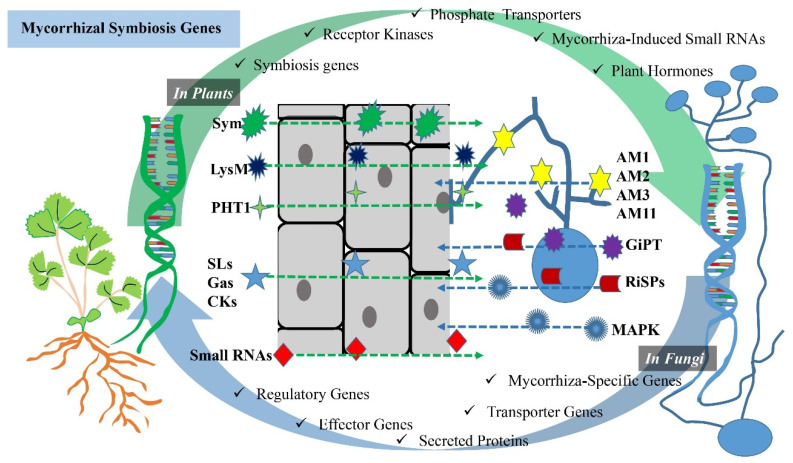
Schematic visualization of genes involved in AM symbiosis. The interaction between plants and AMF involves several genes and molecular components that are crucial for the establishment and maintenance of the symbiotic relationship. While Sym genes, receptor kinases, phosphate (Pi) transporters, mycorrhiza-derived small RNAs and plant hormones in plants are plant-derived genes and components, mycorrhiza-specific genes, transporter genes, secreted proteins, effector genes and regulatory genes are genes of a mycorrhizal fungi origin. These genes and molecular components in both plants and mycorrhizal fungi are essential for the successful establishment and maintenance of mycorrhizal symbiosis, which benefits both partners by improving nutrient exchange and enhancing plant growth.

Mycorrhizal symbiosis involves the interaction between plants and mycorrhizal fungi, and several genes in both partners are crucial for the establishment and maintenance of this symbiotic relationship (Figure 1). Some of the key genes and molecular components involved in mycorrhizal symbiosis are established below.

#### 2.2.1. In Plants

Sym genes (symbiosis genes): these are plant genes that are specifically involved in the establishment and regulation of mycorrhizal symbiosis [36]. They encode various proteins and transcription factors necessary for the recognition of fungal partners, the development of symbiotic structures and the regulation of nutrient exchange [37].

Receptor kinases: plant receptor kinases, such as the LysM receptor kinases, play a role in recognizing fungal signals and initiating the signaling cascade leading to mycorrhizal symbiosis [38,39]. These receptors are essential for the early recognition of mycorrhizal fungi [38].

Phosphate (Pi) transporters: plants upregulate genes encoding phosphate transporters in response to mycorrhizal colonization [40]. These transporters facilitate the uptake of phosphorus from the fungal partner [41].

Mycorrhiza-induced small RNAs: plants produce small RNAs in response to mycorrhizal colonization, which may play a role in regulating the symbiotic interaction [42].

Plant hormones: in general, plant hormones include activities directed towards the development and branching of plants. For example, strigolactones play an important role in stimulating branching activity in plants, contributing to the development of an extensive root system [43]. The gibberellin hormone promotes root elongation and influences plant growth and development [44]. Cytokinins are also involved in the regulation of plant branching. Cytokinins influence the overall architecture of the plant by promoting the growth of lateral buds [45]. Furthermore, various plant hormones, such as strigolactones, gibberellin and cytokinins, are involved in signaling and regulating mycorrhizal symbiosis [7,46,47]. Strigolactones, for example, are known to promote hyphal growth and root colonization by mycorrhizal fungi [6].

#### 2.2.2. In Mycorrhizal Fungi

Mycorrhiza-specific genes: fungi possess genes that are specifically expressed during mycorrhizal symbiosis [48]. These genes are involved in hyphal growth, the establishment of symbiotic structures, like arbuscules, and nutrient exchange [48,49]. For example, Colard et al. [48] reported that AMF-specific AM1, AM2, AM3 and AM11 genes were activated at the pre-symbiotic stage.

Transporter genes: mycorrhizal fungi have transporter genes that code for proteins responsible for the uptake and transfer of nutrients to the plant host [50] (Table 1). For example, phosphate transporters are crucial for delivering phosphorus to the plant [51]. Maldonado-Mendoza et al. [52] revealed that this was thanks to the GiPT gene for *Glomus intraradices.*

Secreted proteins: fungi produce secreted proteins, some of which may be involved in facilitating the interaction with plant roots or modulating plant immune responses [53,54]. For example, Kamel et al.’s [55] study on *Rhizophagus irregularis* revealed that this species had a large number of putative secreted proteins (RiSPs), which could be of great importance in establishing symbiosis.

Effector genes: some mycorrhizal fungi may produce effector proteins that can manipulate plant host defenses or signaling pathways to promote symbiosis [56,57].

Regulatory genes: fungi have genes involved in the regulation of their responses to the plant host and environmental cues [58,59]. These genes help the fungi adapt to different plant partners and environmental conditions [60]. For example, Huang et al. [59] reported that using mitogen-activated protein kinase (MAPK) signaling for the interactions between AMF and apple plant hosts was shown to increase apple drought tolerance.

miRNA: there have also been some miRNAs identified as participants in this regulation, such as miR167, miR394 and miR156 [60].

Understanding the genetic and molecular basis of mycorrhizal symbiosis is an active area of research. These genes and molecular components play a critical role in the formation and maintenance of this beneficial mutualistic relationship, contributing to plant nutrient acquisition, stress tolerance and overall ecosystem health (Table 1).

**Table 1:** AMF-derived genes involved in mycorrhizal symbiosis.

AMF-Induced Genes			References
Mycorrhiza-Specific Genes	AM1, AM2, AM3, AM11	Genes active in the AMF-induced pre-symbiotic stage	[48]
	AM10, AM14, AM15, AM20, AM24, AM25, AM26, AM29	Genes active in the AMF-induced early and mature symbiotic stages	[49]
Transporter Genes	GiPT	AMF-induced plant P transporter genes	[52]
	StPT3		[61]
	OsPT11		[62]
	MtPT4		[63]
	PT11		[49]
	MtZIP5	AMF-induced plant Zn transporter gene	[64]
Secreted Proteins	LbMiSSP7	Secreted proteins regulated by AMF	[65]
	LjCLE19, LjCLE20		[66]
	RiSP		[55]
Effector Genes	RiSLM	AMF-induced effector genes	[67]
	RirG175680, RirG165580, RirG263220, RirG200050, jgi.p|Gloin1|346360, RirG013260, RirG267270, jgi.p|Gloin1|154898, RirG043250, RirG045350, RirG101100, RirG043650, RirG257590, RirG187640, RirG180400, jgi.p|Gloin1|161262		[68]
	PvRxLR18, PvAVH52, PvRxLR28, PvRxLR67	Effector genes against AMF-induced pathogen	[69]
Regulatory Genes	14-3-3	Gene regulating AMF-induced ABA-related signaling pathway	[58]
	MAPK	Genes regulated by AMF to enhance drought tolerance	[59]
	miR167, miR394, miR156		[60]

## 3. Strigolactones

Strigolactones (SLs), a class of plant hormones, have emerged as key regulators of plant growth, development and interactions with the environment. *Striga lutea*’s strigol, the first natural SL, was found as a germination stimulant; as a result, these compounds have subsequently been referred to as SLs [70]. They play a crucial role in various plant processes, such as root development, branching and responses to environmental stresses. These hormones are also known to interact with beneficial soil microorganisms, such as mycorrhizal fungi, to promote nutrient uptake and improve plant health. However, SLs secreted by plants cause the seeds of parasitic plants to germinate. This can cause problems in agricultural areas. In particular, the damage caused to agriculture by witchweed in Africa due to the parasitism on SL signaling is a major issue for farmers in developing countries [71].

### 3.1. Biosynthetic Pathway

According to Matusova et al. [72], SLs are derived from carotenoids, as evidenced by minimal SL accumulation after treatment with the carotenoid biosynthesis inhibitor fluridone in maize plants. The functional role of SLs can be related to their formation and production in response to the needs of the system during evolution [73]. Since it has been discovered that the gene involved in SL production has been reported for many plant species, including algae and bryophytes, it can be hypothesized that these SLs are important molecules that have long persisted in the evolutionary chain [73]. SLs are four-ring (A–D) compounds that change function by the attachment of various groups to the A and B rings [12,15,73,74]. SLs were initially thought to be sesquiterpene lactones, but were later shown to be apocarotenoid derivatives of carotenoid cleavage mediated by carotenoid cleavage dioxygenase (CCDs) enzymes [75]. A member of the CCD family is involved in the production of various apocarotenoid compounds, such as cyclohexenone and mycorradicin [76]. Initial biosynthesis occurs in plastids with the help of three plastid-specific enzymes: D (DWARF)27, CCD7 and CCD8. Carotenoid isomerase D27, carotenoid cleavage dioxygenases CCD7 and CCD8 and cytochrome P450 monooxygenases were identified as SL biosynthesis enzymes through genetic screening for shoot-branching mutants [15]. Furthermore, from mutants with excessive shoot branching, SL biosynthesis genes were found and called more axillary growth (MAX) in *Arabidopsis thaliana* [75], Ramosus (RMS) in *Pisum sativum* [77], decreased apical dominance (DAD) in petunia [78] and dwarf (D) in *Oryza sativa* [79,80].

**Figure 2:**
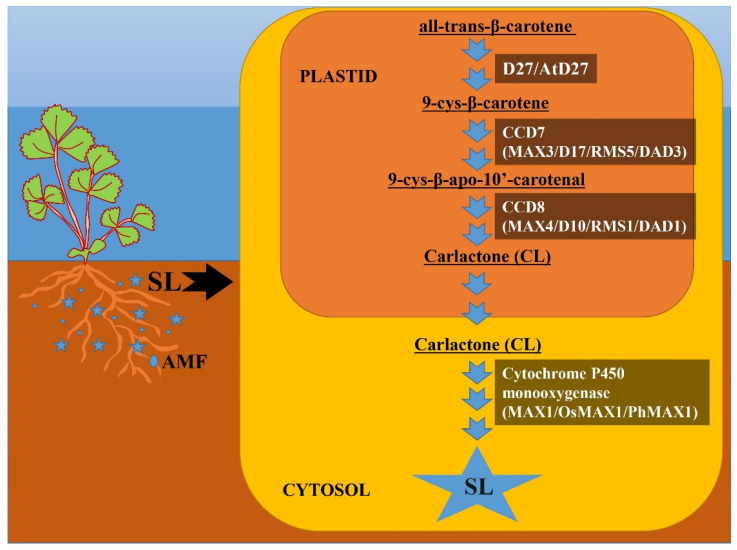
Biosynthetic pathway of strigolactone (SL). This figure depicts the SL biosynthetic route and important enzymes involved in biosynthesis. SL biosynthesis occurs in two distinct compartments: plastid and cytosol. All-trans-β-carotene is converted to carlactone (CL) in plastids via three intermediary stages catalyzed by D27 (At27), CCD7 (MAX3, D17, RMS5, DAD3) and CCD8 (MAX4, D10, RMS1, DAD1), respectively. Carlactone then enters the cytosol, where it is metabolized to several other SLs via cytochrome P450 monooxygenase (MAX1, OsMAX1, PhMAX1) and numerous other unidentified enzymes.

D27 isomerase converts all-trans-β-carotene to 9-cys-β-carotene, and subsequent processes catalyzed by CCD7 and CCD8 convert 9-cys-β-carotene to carlactone (CL) with A- and D-ring structures [15,73]. CL is subsequently oxidized to different SL species by the cytochrome P450 monooxygenase MAX1 or other recently discovered enzymes. Briefly, we show the pathway of SL biosynthesis in Figure 2. Furthermore, not only enzymes downstream of CL but also enzymes upstream of CL may be structurally important for the formation of various SLs in SL biosynthesis. CCD7 and CCD8 carotenoid isomerases convert all-trans-carotene to CL as well as 3-hydroxy-carlactone (3-OH-CL) via zeaxanthin [81]. Although hydroxy-carlactone derivatives are the most common SLs in Arabidopsis [82], their significance for plant growth and development control is unknown.

Recent studies have focused on modifying genes in the Strigolactone biosynthesis pathway using CRISPR/Cas9 gene-editing techniques [83,84,85]. Such genetic modifications can affect the biological processes of plants, such as root development, water use and nutrient uptake, and hence increase their interaction with AMF and abiotic stress tolerance. For example, the carotenoid cleavage dioxygenase 8 (CCD8) genes (SbCCD8a and SbCCD8b), which have been shown to be involved in strigolactone biosynthesis in sorghum, were manipulated by two CRISPR/Cas9-mediated genes and were found to enhance weed control and the activity of beneficial microorganisms [85].

### 3.2. Physiological Functions

Symbiotic interactions: SLs are involved in the establishment of symbiotic associations, particularly with arbuscular mycorrhizal fungi [6,12,14]. They act as rhizosphere signaling molecules, attracting AMF hyphae towards the plant roots and stimulating the branching of fungal hyphae in the soil. Akiyama et al. [86] demonstrated that sesquiterpenes, 5-deoxy-strigol, sorgolactone and strigol extracted from *Lotus japonicus* exudates promoted extended hyphal branching in AMF. A comparable finding was also seen in the synthetic counterpart of sorgolactone [87]. This association enhanced nutrient uptake, particularly phosphorus, by increasing the surface area available for nutrient absorption [6,7,14]. SLs also regulate the development of symbiotic structures, such as arbuscules, which facilitate nutrient exchange between the fungus and the plant [7].

Plant defense responses: SLs contribute to plant defense responses against pathogens and pests [88]. They can prime plants for enhanced resistance by activating defense-related genes and signaling pathways [89]. For example, Nasir et al. [90] found that SLs positively regulated defense against *Magnaporthe oryzae* in rice, and Xu et al. [91] positively regulated defense against root-knot nematodes in tomatoes. SLs can also influence the synthesis of secondary metabolites involved in plant defense mechanisms [92].

Shoot branching: one of the well-known roles of SLs is their influence on shoot branching. SLs act as inhibitors of bud outgrowth, promoting apical dominance and limiting the growth of lateral buds [93]. By suppressing the branching of shoots, SLs ensure the allocation of resources to the main shoot and control the overall architecture of the plant [43]. Manipulating SL levels or signaling can lead to alterations in shoot-branching patterns and can be utilized to enhance branching or promote lateral bud growth when desired [94]. For example, the involvement of SLs in regulating bud growth in *Pisum sativum* and *Arabidopsis thaliana* was demonstrated by Brewer et al. [95].

Overall plant growth and development: SLs have broader effects on plant growth and development. They contribute to various processes, such as seed germination, stomatal closure and responses to environmental stresses [96,97,98]. For example, Ha et al. [99] showed that the genetic modulation of SL content/response could provide a new approach for the development of plants with better environmental stress tolerance. SLs can affect seed dormancy and germination by inhibiting or promoting the germination process, depending on the species and environmental conditions [100]. They also regulate stomatal aperture, reducing water loss through transpiration and aiding in water-use efficiency [97]. SLs influence plant responses to abiotic stresses, such as drought and salinity, by modulating physiological and molecular responses that enhance stress tolerance [99].

## 4. Synergistic Interaction

### 4.1. The Evolution of the Synergistic Interaction

The synergistic interaction between AMF and SLs is an evolutionary phenomenon that has likely developed over millions of years through co-evolutionary processes between plants and fungi. Over time, plants and fungi developed intricate mechanisms to communicate and exchange resources and nutrients, leading to the establishment of symbiotic relationships. SLs, as signaling molecules, have evolved in plants as a means of communication with both beneficial soil fungi and other organisms in the environment [73]. SLs play a crucial role in regulating various plant processes, including root architecture, mycorrhizal associations and defense responses [12,43,88].

The synergistic interaction between AMF and SLs represents the co-optimization of these mutualistic relationships [6,12,14]. AMF have evolved mechanisms to detect and respond to SL signals released by plants, allowing them to colonize roots and establish symbiotic associations more efficiently. In turn, plants have developed mechanisms to enhance SL production and signaling in the presence of AMF, facilitating nutrient exchange and other benefits.

The evolution of this synergistic interaction has likely been driven by the benefits it provides to both plants and AMF. Plants receive improved nutrient uptake, stress tolerance and defense against pathogens, leading to enhanced fitness and survival outcomes. AMF, on the other hand, gain access to plant-derived carbon compounds and a protected niche in the rhizosphere, enabling their growth and reproduction [28].

### 4.2. The Mechanisms Underlying the Synergistic Interaction

The exploration of the mechanisms underlying the synergistic interaction between AMF and SLs involves investigating how these two entities interact at the molecular level to promote plant growth, nutrient acquisition and stress tolerance. In this framework, we list below some of the key molecular mechanisms that contribute to their synergistic interaction.

Receptor-mediated signaling is a key mechanism underlying the synergistic interaction between AMF and SLs. García-Garrido et al. [101] proposed that SLs were a group of terpenoid lactones that functioned as a host-derived signal in plants’ rhizosphere communication with AMF and as an endogenous plant hormone that regulated shoot branching in plants. However, AMF may enhance SL perception and signaling in plants. It is believed that the presence of AMF may affect the expression or activity of SL receptors, leading to greater responsiveness to SL signals [16]. The mechanisms by which AMF enhances SL perception and signaling are not yet fully understood, but we can suggest several possibilities. One possibility is that AMF may produce or release signaling molecules or compounds that enhance the perception of SLs by interacting directly or indirectly with the plant’s SL receptors [7]. These signaling molecules, such as mycorrhization (Myc) components, can modulate the activity or sensitivity of receptors, thus enhancing the plant’s response to SL signals. Another possibility is that AMF may affect the expression or abundance of SL receptors in the plant. By promoting the production or localization of receptors, AMF can increase the plant’s capacity to sense and respond to SLs [12]. This modulation of receptor expression may be mediated by the secretion of specific molecules or enzymes by AMF or by altering plant hormone signaling pathways [12]. In addition, AMF may cause changes in the root system of the plant, such as increased branching or mycorrhizal colonization, which may provide more opportunities for strigolactone receptors to come into contact with SL molecules. This enhanced physical interaction between receptors and SLs may potentiate a signaling response.

Hormonal cross-talk plays a significant role in the synergistic interaction between AMF and SLs, leading to the coordinated regulation of plant growth, nutrient uptake and stress responses [6,7,14]. Both AMF and SLs can modulate hormone signaling pathways in plants, resulting in synergistic effects on plant growth and development.

SLs have been shown to influence the synthesis and signaling of other plant hormones, such as auxins, cytokinins and gibberellins [102]. For example, Hayward et al. [103] report that auxins and SLs interact with each other in a unique feedback loop. This interaction can influence root architecture, lateral root development and mycorrhizal colonization, leading to improved nutrient acquisition and plant performance [104,105]. According to Dun et al. [106], cytokinins and SLs affect the bud-specific gene BRANCHED 1 (BRC1), which encodes a transcription factor that inhibits bud development in *Pisum sativum*. The exogenous administration of SLs, on the other hand, reduces the axillary shoot length both under decapitation [95] and when stimulated by cytokinin [106], providing more support for SL–cytokinin interactions. AMF can produce and modulate the levels of plant hormones. They can influence the synthesis, metabolism and signaling of hormones, such as auxins, cytokinins and abscisic acid [107]. For example, Pons et al. [108] and Mishev et al. [109] emphasized that *Rhizophagus irregularis* modulated phytohormones to interact with host plants or regulate their own development.

The cross-talk between hormonal pathways mediated by AMF and strigolactones allows for the coordinated regulation of plant growth and stress responses [110]. Both AMF and strigolactones are known to enhance plant tolerance to various abiotic stresses, such as drought, salinity and nutrient deficiency [2,105]. It is known that AMF induces the expression of genes related to drought tolerance [111]. Under drought stress, Ruiz-Lozano et al. [111] also found that the SL–ABA interaction was negative in tomato and lettuce plants without mycorrhiza. However, it has been found that the SL–ABA interaction has a positive correlation in stressed mycorrhizal plants [112,113]. However, in the absence of additional stress, low ABA levels have been documented in mycorrhizal plants [112,114,115,116]. A decrease in SL levels has also been observed in plants colonized by AMF [112,117,118,119], which has been proposed to act as a mechanism to prevent over-colonization or as a medium to reduce stress in mycorrhizal plants. Thus, it is obvious that the cross-talk with ABA occurs, at least under adverse circumstances and during the AMF–SL interaction. Further studies, as with other phytohormones, are needed to unravel this intricate relationship during AM symbiosis and stress scenarios.

In the intricate dance of molecular interactions within plant biology, the regulation of gene expression serves as a key orchestrator, guiding various processes critical to plant growth and development. In the fascinating interplay between AMF and SLs, three distinct modes of gene expression regulation come to light: “SL Regulation”, “AMF Regulation” and “Coordinated Regulation”.

*SL regulation of gene expression*: SLs can modulate the expression of genes associated with various processes in plants. For example, Marro et al. [120] reported that SLs modulated the expression of important regulatory genes in the phosphate (P) and nitrate (N) signaling pathways, such as PHO_2_ and NIGT_1_/HHO integrators. They play a significant role in regulating lateral root development, promoting the initiation and elongation of lateral roots [121]. SLs can also influence AMF colonization through the genes they can modulate. For example, in rice plants, SMAX1 has been shown to be a suppressor of AM symbiosis, negatively regulating its colonization and the transcription of important signaling components and conserved symbiosis genes [122]. On the other hand, SLs have been shown to modulate the D14 gene, resulting in a high rate of AMF colonization [123]. Additionally, SLs can regulate stress-responsive genes, enhancing the plant’s ability to cope with abiotic and biotic stresses [110]. In general, SLs regulate the activation of specific pathways and molecular responses in plants by modulating gene expression. These include the pathways related to nutrient acquisition, hormonal regulation and defense mechanisms. The influence of SLs on gene expression contributes to the synergistic effects with AMF, leading to coordinated plant growth and stress responses.

*AMF regulation of gene expression*: AMF can induce the expression of specific genes in plants. For example, AMF affects the transmembrane transport of water by modulating AQP genes encoding aquaporin water-channel proteins located in cell membranes, suggesting that AMF enhances drought tolerance in plants [124,125,126]. In addition, drought treatments did not alter the expression of the AQP protein GintAQP1 (in *Glomus intraradices*) but induced the expressions of GintAQPF1 (in *G. intraradices*) and GintAQPF2 (in *G. intraradices*), while in the AQP protein RcAQP3 (in *Rhizophagus clarus*) it was expressed in the intraradical hyphae to transport water [127,128,129,130]. There is a small amount of evidence that AMF also contribute to the reduction in oxidative stress in the antioxidant system. Some studies have cloned antioxidant genes from AMF involved in reducing ROS accumulation. These genes have been reported as the GmarCuZnSOD gene in *Gigaspora margarita* [131] and GintMT1 [132], GintGRX1 [133], GintPDX1 [134] and GintSOD1 [135] in *G. intraradices*. In general, AMF have a strong ability to cope with damage by regulating molecular responses to tolerate different stressors.

*Coordinated regulation of gene expression*: the combined effect of AMF and SLs results in a coordinated regulation of gene expression, leading to synergistic effects on plant growth and resistance [136]. Moreover, these two partners activate complementary pathways that promote plant growth, nutrient uptake and stress tolerance by targeting different sets of genes. For example, SL production and AMF colonization have been reported to help plants cope with salt stress by inducing the expression of genes involved in ABA biosynthesis [112]. However, the cross-talk between the molecular mechanisms affected by AMF and strigolactones leads to a coordinated response, ensuring optimal plant performance. For example, Ruiz-Lozano et al. [113] reported that SLs induced AMF colonization, which in turn affected drought-related genes, making tomato plants more resistant to drought stress. At the same time, the expression of genes involved in nutrient uptake and transport may be synergistically regulated by the combined action of AMF and SLs, maximizing nutrient uptake efficiency [137]. Similarly, the activation of defense-related genes in response to AMF can be enhanced by SLs, leading to increased plant resistance to pathogens and pests [138]. Overall, the coordinated regulation of gene expression by AMF and SLs contributes to the overall improvement of plant growth, nutrient utilization and stress tolerance.

## 5. Significance of the Interaction

### 5.1. Influence on Plant–Microbe Symbiosis and Rhizosphere Dynamics

The interaction between AMF and SLs has a great influence on plant–microbe symbiosis and rhizosphere dynamics. This interaction shapes the microbial community composition, promotes beneficial interactions and modulates the biochemical and physical properties of the rhizosphere [139].

SLs play a crucial role in initiating and enhancing the colonization of AMF, leading to the establishment of functional mycorrhizal symbiosis [7,14]. This symbiotic association benefits both the plant and the fungi, promoting nutrient uptake, stress tolerance and overall plant performance and crop yield [6,140,141,142]. Furthermore, this interaction influences the composition and diversity of the microbial community in the rhizosphere. SLs contribute to the regulation of microbial interactions by modulating the production of secondary metabolites and influencing microbial communication systems [143]. This modulation of the microbial community promotes a favorable rhizosphere environment, facilitating beneficial plant–microbe interactions and reducing the damages of stress factors. For example, Mostofa et al. [144] suggested that SLs were involved in regulating the biosynthesis of secondary metabolites, such as flavonoids, to enhance plant protection against osmotic stresses.

The AMF–SL interaction affects the biochemical and physical properties of the rhizosphere, creating a dynamic and conducive environment for plant growth [145]. AMF enhances soil aggregation, improving soil structure and porosity [146]. This results in increased water infiltration, nutrient availability and root exploration in the rhizosphere. SLs influence the secretion of root exudates, altering the chemical composition of the rhizosphere and influencing microbial interactions. These changes in rhizosphere dynamics have implications for nutrient cycling, carbon sequestration and overall soil health [147,148]. Moreover, this interaction promotes resource exchange between plants and microbes in the rhizosphere [86]. AMF facilitates nutrient uptake by extending their hyphae into the soil, increasing the nutrient-absorbing surface area. In return, the plants provide carbon compounds through root exudates, which serve as an energy source for AMF [149]. This reciprocal exchange of nutrients and carbon compounds enhances nutrient cycling, promotes soil fertility and supports sustainable agricultural practices.

A notable component influencing rhizosphere dynamics is glomalin, a glycoprotein produced by AMF [150]. Glomalin contributes to soil structure stability, enhances the water retention capacity and aids in carbon sequestration [151]. Its presence in the soil is associated with the mycorrhizal hyphae, and the binding of glomalin to soil particles creates aggregates that improve soil structure [151]. This, in turn, supports water movement, nutrient availability and the overall health of the rhizosphere. The intricate interplay between AMF, SLs and glomalin (a tripartite interaction) highlights the multifaceted nature of plant–microbe interactions in shaping the rhizosphere environment and promoting sustainable soil management practices [152]. In this tripartite interaction, the role of SLs is also important in this process because these signaling molecules attract AMF, encouraging it to establish a symbiotic relationship with the roots. This interaction involves plants releasing SLs through their roots, resulting in the withdrawal of AMF and transporting glomalin along with the roots.

### 5.2. The Effects against Biotic Stresses

**Figure 3:**
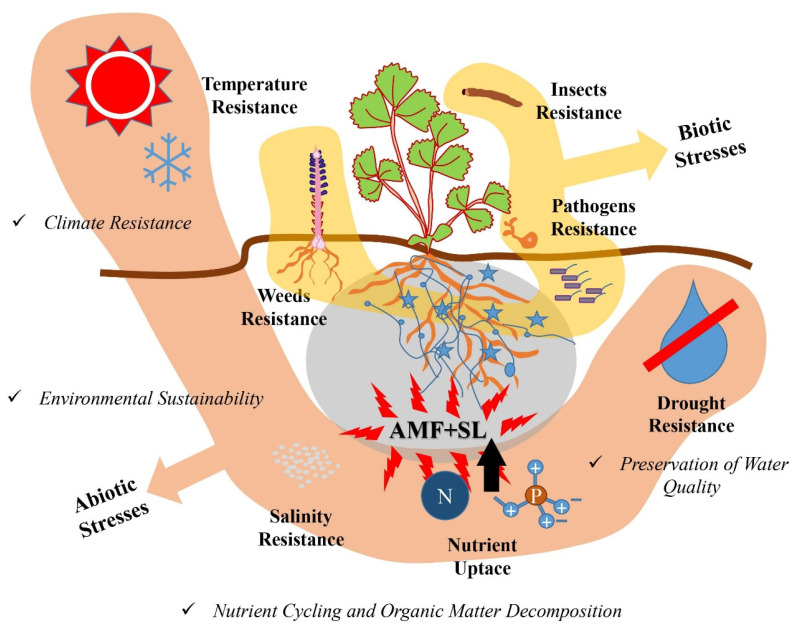
Effects of AMF–SL interaction on biotic and abiotic stress factors. The interaction between AMF and SLs plays an important role in enhancing plant resistance to abiotic and biotic stresses. These synergistic partners can enhance plant resistance to abiotic stresses, such as drought, salinity, temperature extremes and nutrient deficiencies, and biotic stresses, such as disease, pests and weeds. Furthermore, these partners can significantly benefit sustainable agro-ecosystems, such as environmental sustainability, climate resistance, nutrient cycling, organic matter decomposition and the preservation of water quality.

The interaction between AMF and SLs has important effects against pathogens and weeds [153] (Figure 3). These effects on subsurface interactions might be caused by different mechanisms. For example, Cordier et al. [154] demonstrated that mycorrhizae compete with other pathogens for colonization sites by the complete exclusion of *Phytophthora* from arbusculated cells. Colonization by AMF can lead to changes in the quality and quantity of root exudates [155,156,157,158,159]. For example, Lendzemo et al. [117] and López-Ráez et al. [160] suggested that AMF increased SL production in the early stage of colonization; in later stages, both SL and salicylic acid production were suppressed, whereas jasmonates biosynthesis was increased. This root exudation modulated by AMF also leads to the effect of mycorrhizae on plant interactions with parasitic plants. López-Ráez et al. [119], for example, demonstrated that mycorrhiza reduced the occurrence of root parasite plants in *Orobanchaceae*, including the genera *Striga*, *Orobanche* and *Phelipanche*. This opens the possibility of employing AMF to manage parasitic weeds where traditional methods have failed.

The root exudation altered by the AMF–SL interaction may also directly affect microbial pathogens and nematodes. For example, exudates from mycorrhizal tomatoes temporarily paralyze nematodes and generally reduce their penetration into mycorrhizal tomato roots [161]. However, systemic root protection against oomycetes and bacterial pathogens in tomatoes [154,162,163,164], against fungal pathogens in barley [164] and against nematodes in banana and grapevine [165] has been confirmed. It was also shown by Jung et al. [9] that plant defense mechanisms regulated by jasmonate as a result of the AMF–SL interaction restricted the development of necrotrophic pathogens and the performance of phytophagous insects. In addition to their activity as signaling molecules in the rhizosphere, SLs also play a role in signaling within the plant by regulating shoot and root morphologies. It has been proposed that SLs, in conjunction with auxins, encourage lateral root expansion, allowing the root system to reach new regions of the soil where phosphate may be present [166]. SL-mediated changes in root architecture may alter the dynamics of some pathogen infections, but direct evidence of such a correlation is lacking. However, SLs may alter the dynamics of pathogen infections through AMF or by enhancing plant development.

### 5.3. The Effects against Challenging Environmental Conditions

The interaction between AMF and SLs also plays a significant role in enhancing plant resistance against abiotic stresses (Figure 3). Abiotic stresses, such as drought, salt, extreme temperatures and nutrient deficiencies, can have detrimental effects on plant growth and productivity [167]. The AMF–SL interaction may help plants to cope with abiotic stresses.

*Drought Stress:* AMF and SLs contribute to improved drought tolerance in plants. AMF enhance the plant’s ability to cope with drought by improving water-use efficiency and water uptake [2,168,169]. They help plants access water in deeper soil layers through extensive mycelial networks or by increasing root biomass and root hydraulic conductivity. SLs play a role in regulating stomatal closure and transpiration, reducing water loss and enhancing water-use efficiency [170,171]. The action of AMF and SLs enables plants to withstand periods of water scarcity, maintain cellular hydration, and sustain growth and productivity. It may do so by potentiating SL responses of AMF [113]. Indeed, phytohormones, such as SLs, are involved in plant water stress regulation [144]. Furthermore, Huang et al. [172] showed that the overexpression of MdIAA24, one of the SL synthesis genes in apples, favorably affected arbuscule formation and helped the plant to cope with drought stress.

*Salt stress:* salinity stress negatively affects plant growth and development by impairing water uptake and causing ion imbalances [173]. The interaction between AMF and SLs helps plants cope with salinity stress. AMF improve salt tolerance by promoting ion homeostasis and reducing the uptake of toxic ions, such as sodium [174]. They enhance nutrient uptake efficiency, especially for essential nutrients, like potassium, which can counterbalance the effects of sodium toxicity [175]. SLs regulate the expression of genes involved in ion transport and osmotic adjustment, contributing to salt stress mitigation [176]. Furthermore, Ha et al. [99] suggested that the genetic modulation of SLs may provide a new approach for the development of plants with better tolerance to salt stress. The combined action of AMF and SLs improves salt tolerance, allowing plants to maintain cellular integrity, minimize osmotic stress and sustain growth under saline conditions. For example, Kong et al. [177] showed that the interaction of AMF and SL enhanced salt stress tolerance by maintaining the cellular integrity in *Sesbania cannabina* seedlings. Furthermore, Aroca et al. [112] suggested that, under salt stress conditions, lettuce plants increased SL production to promote the formation of AMF colonization to cope with salt stress.

*Temperature stress:* extreme temperatures, both cold and heat, can disrupt plant growth and development [178]. In the current context of climate change, this is not only a thread for agricultural yield, but also for natural ecosystems, specially forests [179]. AMF and SLs help plants mitigate the adverse effects of temperature stress. AMF enhance plant thermotolerance by inducing the production of heat shock proteins, antioxidants and other protective compounds. For example, Maya and Matsubara [180] suggested that, under heat stress conditions, AMF improved plant growth by increasing nutrient and water uptake and increased the activity of antioxidant enzymes in cyclamen plants. Furthermore, AMF improved the water-use efficiency and photosynthetic rate in wheat and maize plants under heat stress conditions [137,181]. SLs promote tolerance to temperature stress by contributing to the regulation of temperature stress-responsive genes and the modulation of hormone signaling pathways. For example, GR24, an analog of SLs, has positive effects on the elongation of crown roots and the number of root cells in *Festuca arundinacea* under heat stress conditions, with changes observed in the expression patterns of cell division and cell cycle-related genes in the root tips, such as cyclin-D2 (CycD2), proliferating cell nuclear antigen (PCNA) and cyclin-dependent kinase B (CDKB) [182]. Furthermore, Tsuchiya et al. [183] showed that SLs were upregulated in *Arabidopsis thaliana* mutants (max1, max3) throughout heat stress. Moreover, the exogenous application of the SL analog GR24 increased the expression of the ABA catabolic gene (CYP707A1), suggesting that seeds germinated even under heat stress conditions [184]. The combined action of AMF and SLs helps plants withstand temperature extremes, maintain physiological functions and minimize damage caused by heat or cold stresses. Given that SLs serve as host identification signals for AMF in this combined interaction, it is possible that SLs act as a “call for help” signal, triggering a positive feedback loop for AMF colonization that increases the plant’s tolerance to abiotic stresses, such as heat stress [185].

*Nutrient deficiencies:* nutrient deficiencies can limit plant growth and productivity. The interaction between AMF and SLs improves nutrient acquisition and utilization efficiency, enhancing plant tolerance to nutrient deficiencies. AMF enhance the availability and uptake of essential nutrients, especially phosphorus, which is often limited in soils [186,187]. They facilitate the exploration of a larger volume of soil, accessing nutrients beyond the root’s reach [188]. SLs contribute to the regulation of nutrient-responsive genes and hormonal cross-talk, optimizing nutrient utilization and improving plant resistance to nutrient deficiencies [189]. On the other hand, SLs have recently been found to control plant response or perception to phosphorus-limited conditions [190]. Surprisingly, some studies have suggested that SLs may have a function in rice plant root development in the absence of P and N [190]. This may suggest that plants increase their tolerance to nutrient deficiency stress through SLs. The combined action of AMF and SLs ensures efficient nutrient uptake, minimizing the detrimental effects of nutrient limitations on plant growth and productivity. Several studies have revealed that SLs play an important role in adaptive responses to P and N deficiencies due to increased SL levels in plant roots [191,192,193]. For example, SLs promote a symbiotic connection to AMF by stimulating hyphae branching and modify shoot architecture by reducing tiller bud development to respond to N- or P-deficiency conditions [193,194]. Furthermore, Mitra et al. [6] suggest that SLs drives the development of roots and the symbiotic relationship of AMF that enhances the uptake of various nutrients, mainly phosphorus, from the rhizosphere, which in turn makes the plant more resistant to nutrient deficiency. Recently, it has been shown that potassium can also be a usual limiting factor in agricultural soils [195]. The influence of AMF on potassium nutrition is a largely unexplored topic [196], and whether the SL–AMF interaction regulates the acquisition of this major nutrient is unknown at present. This topic should be investigated in the future, not only for the gaining of basic knowledge, but for the fact that modulating potassium transport is a standard strategy to overcome abiotic stress in plants [197].

### 5.4. The Effects on Sustainable Agro-Ecosystems

The interaction between AMF and SLs can significantly benefit the sustainable agro-ecosystem (Figure 3). The most important benefits of this interaction are listed below.

*Environmental sustainability:* the utilization of AMF and SLs supports environmentally sustainable agricultural practices. By enhancing nutrient uptake efficiency and nutrient cycling, this application reduces nutrient losses and minimizes the environmental pollution caused by excessive fertilizer use [198]. The suppression of diseases and pests through the interaction between AMF and SLs reduces the reliance on chemical pesticides, preserving beneficial organisms and promoting ecological balance [199,200,201]. This application contributes to the conservation of soil health, biodiversity and overall ecosystem sustainability [140,202,203].

*Climate resistance:* the interaction between AMF and SLs enhances the resistance of crops to abiotic stresses, such as drought, salinity and temperature extremes [112,140,172,185,193]. AMF improve water-use efficiency, salt tolerance and heat/cold stress responses, enabling plants to better withstand adverse environmental conditions [174,180,187]. SLs modulate hormonal signaling pathways and defense responses, reinforcing plant resistance to abiotic stresses [171,182]. This applications helps mitigate the negative impacts of climate change on crop production, ensuring food security and agricultural sustainability [204,205].

*Nutrient cycling and organic matter decomposition:* AMF and SLs contribute to nutrient cycling and organic matter decomposition in the soil. The symbiotic associations formed by AMF enhance nutrient uptake and transfer in the rhizosphere, allowing for efficient nutrient cycling between plants and microbes. These fungi can mineralize organic compounds and access organic forms of nutrients, making them available to plants [148]. SLs, through their influence on root exudates, contribute to organic matter decomposition by stimulating the activity of microbial decomposers [206,207]. This enhances nutrient release from organic residues, further improving nutrient availability for plant uptake [208].

*Preservation of water quality:* by enhancing nutrient use efficiency and minimizing nutrient losses, the interaction between AMF and SLs contributes to the preservation of water quality [140,209]. The excessive application of chemical fertilizers can result in nutrient runoff, leading to the eutrophication of water bodies and the disruption of aquatic ecosystems. The use of AMF and SLs helps reduce nutrient losses from agricultural fields, preventing water pollution and maintaining water quality [6,171,210]. This application supports sustainable water resource management and the conservation of aquatic biodiversity.

## 6. Agricultural Applications

### 6.1. Application Methods

The use of AMF and strigolactones in sustainable agriculture may involve diverse application methods depending on the specific context and the desired outcomes. A description of the possible application techniques, including the use of synthetic analogs of strigolactones (SASLs), is presented below. However, before these application methods are used, SASLs must be solubilized with different solvents.

**Figure 4:**
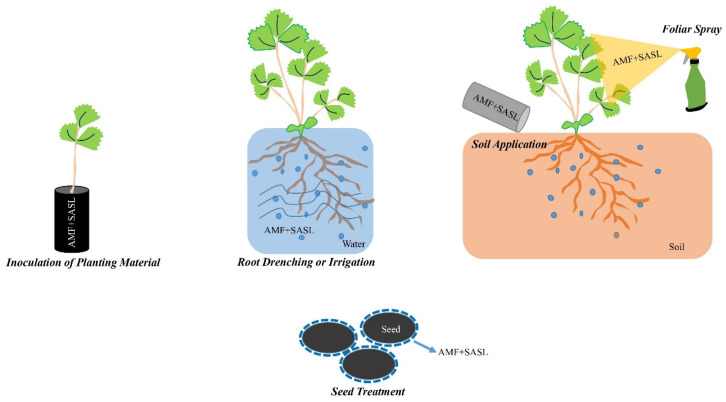
Application methods of AMF and strigolactones in agricultural fields. The use of AMF and strigolactones in sustainable agriculture can involve various application methods depending on the specific context and desired results. These application methods are used in the form of synthetic analogs of strigolactones (SASLs). Preparations prepared as a result of synergistic effects between AMF and SASLs can be applied in agricultural fields by methods, such as seed treatment, soil application, root drip or irrigation, foliar spraying and inoculation of planting material.

*Seed treatment:* SASLs can be included in seed treatments alongside AMF [211] (Figure 4). By coating or inoculating the seeds with a formulation containing AMF and SASLs, the seeds are primed for enhanced root development and the establishment of mycorrhizal associations. The presence of SASLs can stimulate the release of plant root exudates that attract beneficial fungi, further promoting symbiotic interactions and nutrient uptake [212].

*Soil application:* SASLs can be incorporated into soil applications along with AMF [213] (Figure 4). This method involves applying a mixture of AMF, SASLs, and organic amendments to the soil during land preparation or at specific crop growth stages. SASLs, together with AMF, can enhance the colonization of plant roots by mycorrhizal fungi, leading to improved nutrient acquisition and soil health [147].

*Root drenching or irrigation:* SASLs can be included in root drenches or irrigation systems along with AMF [91] (Figure 4). The mixture of AMF and SASLs is diluted in water and applied directly to the root zone or through irrigation systems. This method ensures direct contact between the AMF, SASLs and plant roots, facilitating the establishment of mycorrhizal associations and promoting plant growth, nutrient uptake and stress tolerance [91,113].

*Foliar spray:* while the direct application of SASLs as a foliar spray may not be as common, it can be used alongside AMF in some instances [214] (Figure 4). The mixture of AMF and SASLs is diluted in water and sprayed onto the leaves of plants. Although the primary mode of action of strigolactones is through the roots, the foliar application of SASLs can still have indirect effects on plant growth, hormonal signaling and possibly stimulate root development [215].

*Inoculation of planting material:* SASLs can be incorporated into the inoculation of planting material alongside AMF (Figure 4). The planting material, such as tree seedlings or transplants, can be treated with a mixture of AMF and SASLs prior to transplantation. This treatment primes the roots of the seedlings or transplants for beneficial mycorrhizal associations, enhancing nutrient uptake and promoting plant establishment and growth [216,217,218].

Suicidal germination: a recently promising option in the fight against Striga. This strategy refers to reducing the seed bank in infested soils by applying synthetic germination stimulants in the absence of the host [219,220]. Indeed, Striga is one of the greatest global biotic threats to agriculture, especially in sub-Saharan Africa, causing severe yield losses in cereals [221]. A series of SASLs can be used to develop a protocol for implementing the suicidal germination strategy for combating Striga, GR5 and GR7 [222], Nijmegen-1 [223], analogs derived from ketones and cyclic keto enols [224,225] and analogs recently developed, derived from methyl phenlactonoates [226]. Its co-administration with AMF may further enhance the efficacy of this strategy.

### 6.2. Agricultural Application Areas

**Figure 5:**
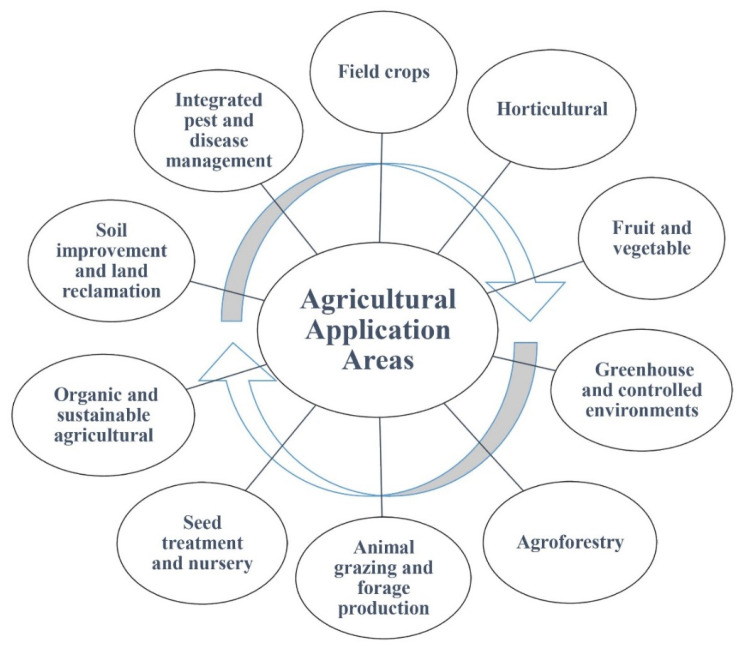
Schematic view of the utilization of AMF and SLs in various agricultural application areas.

Agricultural applications can demonstrate the versatility and potential of using AMF and SLs in various agricultural sectors to promote sustainable and environmentally friendly farming practices, increase crop productivity and effectively manage pests, diseases and soil health (Figure 5).

Field crop applications refers to the use of AMF and SLs in the cultivation of large-scale field crops, such as cereals, oilseeds and fiber crops to improve nutrient uptake, increase yield, improve stress tolerance and manage diseases and pests [227,228,229,230]. Horticultural applications include the application of AMF and SLs in the cultivation of horticultural crops, including fruit, vegetables and ornamentals, to promote healthy plant growth, increase nutrient uptake, improve crop quality and reduce disease and pests [231,232,233]. Applications in fruit and vegetable production focus on the application of AMF and SLs and aim to increase the yield, improve fruit quality, increase nutrient uptake and manage disease and pests to ensure optimum crop production [232,234]. However, applications in greenhouse and controlled environments refer to the use of AMF and SLs in greenhouse and controlled environments where the environmental conditions are closely regulated [235,236]. These applications aim to optimize plant growth, improve nutrient utilization, increase stress tolerance and manage disease and pests in closed cropping systems.

The application of AMF and SLs in agroforestry systems promotes beneficial interactions between trees and crops, increases nutrient cycling, improves soil health and reduces disease and pests, contributing to sustainable and diversified agricultural practices [237,238]. The application of AMF and SLs in pasture and forage systems important for animal grazing and forage production can be important for pasture and forage management [239,240]. These practices aim to improve nutrient uptake, increase forage quality and quantity, increase plant resistance to grazing and manage disease and pests for optimum animal nutrition.

Seed treatment and nursery applications include the treatment of seeds and application of AMF and SLs in nurseries to improve seed germination, seedling growth, root development and overall plant health [6,241]. These practices provide a strong foundation for healthy and strong plants. It is also important to consider the use of AMF and SLs in organic and sustainable agricultural practices. These practices support the principles of organic farming by promoting nutrient cycling, improving soil health, increasing plant resilience and reducing the dependence on synthetic inputs [140]. AMF and SLs can be included in soil improvement and land reclamation projects, and can improve soil quality, restore degraded land, enhance nutrient cycling and establish vegetation cover in areas affected by pollution, mining or other forms of land degradation [160,242].

Integrated pest and disease management focuses on an integrated approach to managing pests and diseases by combining the application of AMF and SLs with other control measures, such as biological control agents, cultural practices and crop rotation [13,243,244]. This approach aims to minimize the dependence on chemical pesticides, increase plant resistance and promote a balanced and sustainable pest and disease management strategy.

## 7. Future Directions and Conclusion

### 7.1. Unexplored Aspects and Knowledge Gaps in the Field

There is still much to be uncovered regarding the precise molecular mechanisms underlying the interaction between AMF and SLs [245]. The investigation of specific signaling pathways, transcriptional regulation and related gene expression patterns will provide greater insights into this complex interaction. Furthermore, the cross-interactions between AMF–SLs and other signaling pathways, such as hormone signaling and defense responses, remain relatively unexplored [246]. Investigating how these pathways interact and synergistically contribute to plant growth promotion and stress tolerance will improve our understanding of the broader regulatory networks involved.

The influence of environmental factors, such as soil properties, climatic conditions and agronomic practices, on the efficiency of the AMF–SL interaction requires further investigation [247]. Understanding how these factors modulate the interaction will enable the development of tailored strategies for different agroecosystems and environmental conditions.

Different crops may respond differently to the AMF–SL interaction due to differences in their genetic backgrounds, root architectures and physiological properties [141]. Investigating product-specific responses and identifying the optimal application strategies for different crops will increase the practical application and scalability of this interaction. However, the long-term effects of the AMF–SL interaction on soil health, microbial communities and ecosystem dynamics are still poorly understood. An investigation of the potential long-term effects, both positive and negative, will contribute to a comprehensive assessment of the sustainability and environmental impacts of using AMF and SLs in agriculture.

Addressing these unexplored aspects and knowledge gaps through further research will improve our understanding of the AMF–SL interaction and its potential applications in sustainable agriculture. By shedding light on these areas, researchers can focus their efforts on filling in these knowledge gaps and generate valuable information for practical applications and future advances in the field.

### 7.2. Future Research Directions to Advance the Understanding of the AMF–SL Interaction

Future research directions to advance the understanding of the AMF–SL interaction involve several key areas that can contribute to enhancing our knowledge and practical applications [168,198,248,249]. In this framework, some potential research directions could be as follows:

*Mechanistic studies:* conducting in-depth mechanistic studies to unravel the molecular and biochemical processes involved in the interaction between AMF and SLs. This may involve investigating specific genes, proteins and metabolic pathways that mediate the interaction and understanding how they contribute to plant growth promotion, nutrient uptake and stress tolerance. Their impact on potassium nutrition merits special attention.

*Omics approaches:* employing high-throughput omics technologies, such as genomics, transcriptomics, proteomics and metabolomics, to gain a comprehensive understanding of the global changes occurring in plants during the AMF–SL interaction. These approaches can provide valuable insights into the regulatory networks, metabolic pathways and molecular responses underlying the synergistic effects.

*Genetic manipulation:* using genetic manipulation techniques, such as gene knockout or overexpression, to investigate the specific roles of key genes involved in the AMF–SL interaction. This can help identify the essential components and regulatory elements that drive the beneficial effects observed and potentially enhance the efficacy of the interaction.

*Crop-Specific Studies:* Conducting crop-specific studies to assess the efficacy and practical applicability of the AMF–SL interaction across different crop species. This can provide insights into the variability of responses and help tailor application strategies for specific crops, taking into account their genetic backgrounds, root architectures and physiological characteristics.

*Environmental impact assessment:* conducting comprehensive studies to assess the environmental impacts and sustainability of using AMF and SLs in agriculture. This may involve evaluating the effects on soil health, microbial communities, ecosystem dynamics and potential ecological risks associated with the long-term use of these approaches.

*Field trials and validation:* conducting large-scale field trials and validation studies to evaluate the efficacy, practicality and economic viability of using AMF and SLs in real-world agricultural settings. These studies can provide practical insights into the application methods, dosage, timing and compatibility with existing agricultural practices.

*Multi-disciplinary approaches:* encouraging interdisciplinary collaborations between plant biologists, microbiologists, agronomists, ecologists, soils specialists, formulation experts and bioinformaticians to foster a holistic understanding of the AMF–SL interaction. This can facilitate the integration of diverse expertise, data and methodologies to address complex research questions and bridge the gap between fundamental knowledge and practical applications.

*Future perspectives of AMF inoculation effectiveness:* the effectiveness of AMF inoculation may offer a number of possible perspectives in the future. For example, it can improve plant growth and soil fertility. This can contribute to sustainable agricultural practices. It may have the potential to minimize environmental impacts by reducing the use of chemical fertilizers and pesticides that are harmful to the soil. It can positively affect food production and safety. It can be a strategy for coping with climate change and changing environmental conditions, as well as increasing biodiversity among natural plant communities and ecosystems.

*Strategies to improve AMF viability in formulation and shelf-life utilization:* the preservation of AMF viability and shelf life is essential for their effective use in agricultural applications. The mycorrhizal inoculum formulation should be developed as a suitable carrier material that provides a protective environment for AMF that allows an easy application. Common carriers include inert materials, such as sterilized soil, vermiculite, perlite or clay-based granules. It may also be considered to add protective additives, such as organic matter, humic substances or microbial stabilizers, to the formulation. An optimum moisture level must be maintained in the formulation to avoid drying out or an excessive water content, which can damage the AMF.

*Future strategies for the use of AMF and SLs:* in the future, strategies can be developed to use natural microbial and plant signaling molecules, such as AMF and SLs, to focus on sustainability, productivity and environmental protection goals in agriculture and ecosystem management. These strategies include strengthening the symbiotic relationship of AMF with plants and increasing soil fertility by enhancing phosphorus uptake through the use of SLs, coping with climate change by reducing soil erosion and improving the water retention capacity, improving plant nutrient value and developing a resistance to malnutrition, reducing the use of chemical fertilizers and pesticides through biocontrol strategies and natural nutrient delivery. In addition, factors, such as farmer training, scientific communication and the integration of technological developments are also important for the successful implementation of these strategies.

By pursuing these future research directions, we can expand our understanding of the AMF–strigolactone interaction and unlock its full potential for sustainable agriculture. These research efforts will contribute to the development of innovative strategies for enhancing crop productivity, nutrient use efficiency and stress resilience, while minimizing the environmental impacts.

### 7.3. Concluding Remarks

The exploration of the molecular mechanisms underlying the AMF–SL interaction revealed the intricate signaling pathways, gene expression regulation and hormonal cross-talk involved. The induction of SL production by AMF, enhanced colonization, and establishment of AMF in the presence of SLs and the modulation of plant defense responses and nutrient acquisition were key mechanisms contributing to the synergistic effects observed. The significance of this interaction lies in its potential to improve crop productivity, nutrient use efficiency and soil health. It also offers opportunities for reducing chemical inputs and minimizing the environmental impact of agricultural practices.

Partners, such as AMF and SLs, have a potential importance for promoting the sustainability and productivity of agriculture. However, the vaccination efficacy of these partners and the formulations and procedures developed to maintain vigor and viability have not yet been addressed in detail. In particular, further research should be conducted on the inoculation efficacy of AMF and SASLs. This can help us to understand how effective the use of these partners in agricultural fields is and how they contribute to plant development. Moreover, original strategies on how to use the knowledge on AMF and SLs to tackle agricultural challenges should also be identified.

## Data Availability

Not applicabe.
